# A cAMP-biosensor-based assay for measuring plasma arginine–vasopressin levels

**DOI:** 10.1038/s41598-024-60035-4

**Published:** 2024-04-24

**Authors:** Kosuke Doi, Kouki Kawakami, Tatsuya Ikuta, Asuka Inoue

**Affiliations:** 1 Research and Development Section, Diagnostics Division, YAMASA Corporation, Choshi, Chiba 288-0056 Japan; 2 Graduate School of Pharmaceutical Sciences, Tohoku University, Sendai, Miyagi 980-8578 Japan

**Keywords:** Biomarkers, Endocrinology, Medical research

## Abstract

Arginine–vasopressin (AVP), a cyclic peptide hormone composed of nine amino acids, regulates water reabsorption by increasing intracellular cyclic adenosine monophosphate (cAMP) concentrations via the vasopressin V2 receptor (V2R). Plasma AVP is a valuable biomarker for the diagnosis of central diabetes insipidus (CDI) and is commonly measured using radioimmunoassay (RIA). However, RIA has several drawbacks, including a long hands-on time, complex procedures, and handling of radioisotopes with special equipment and facilities. In this study, we developed a bioassay to measure plasma AVP levels using HEK293 cells expressing an engineered V2R and a cAMP biosensor. To achieve high sensitivity, we screened V2R orthologs from 11 various mammalian species and found that the platypus V2R (pV2R) responded to AVP with approximately six-fold higher sensitivity than that observed by the human V2R. Furthermore, to reduce cross-reactivity with desmopressin (DDAVP), a V2R agonist used for CDI treatment, we introduced a previously described point mutation into pV2R, yielding an approximately 20-fold reduction of responsiveness to DDAVP while maintaining responsiveness to AVP. Finally, a comparison of plasma samples from 12 healthy individuals demonstrated a strong correlation (Pearson's correlation value: 0.90) between our bioassay and RIA. Overall, our assay offers a more rapid and convenient method for quantifying plasma AVP concentrations than existing techniques.

## Introduction

Arginine–vasopressin (AVP) is a cyclic peptide hormone consisting of nine amino acids, with a disulfide bond between Cys1 and Cys6. It is produced in the hypothalamus and is released from the posterior pituitary gland. AVP regulates various physiological functions by activating its cognate G-protein-coupled receptors (GPCRs)^1^. Among the three AVP receptors, the vasopressin V2 receptor (V2R) is mainly expressed in renal tubular epithelial cells and is responsible for water reabsorption via Gs-mediated cyclic adenosine monophosphate (cAMP) production^2^.

One of the most recognized disorders involving AVP and V2R is diabetes insipidus, and the measurement of blood AVP levels is essential for diagnosing the disease. Diabetes insipidus can be broadly categorized into central diabetes insipidus (CDI) and nephrogenic diabetes insipidus (NDI)^3^. CDI is caused by a complete or partial deficiency of AVP, resulting in blood AVP levels lower than those in healthy individuals, and clinical symptoms, such as polyuria and polydipsia^4^. In contrast, NDI is caused by the impaired sensitivity to AVP in the kidney, despite normal blood AVP levels in the patients. CDI and NDI require distinct therapeutic strategies, and differentiating between these two diseases is crucial for effective treatment^5^. Accordingly, determining the blood AVP level is instrumental in the diagnosis of CDI. This is typically determined using radioimmunoassay (RIA)^6–8^. However, the RIA method is ill-suited for routine diagnosis because of the need for intricate handling of radioisotopes and specialized instruments, complicated procedures, and lengthy hands-on time that can extend for several days. Given these challenges, there is a pressing need for straightforward and rapid assays.

We hypothesized that a biosensor-based cAMP accumulation assay could be an alternative method for measuring blood AVP levels. A recent study reported the successful application of a similar biosensor-based assay to measure levels of thyroid-stimulating autoantibodies (TSAbs) using cells co-expressing the thyroid-stimulating hormone receptor (TSHR) and a cAMP biosensor^9,10^. In this assay, the cAMP biosensor measured the increase in intracellular cAMP levels triggered by TSHR activation promoted by blood TSAbs. Notably, the assay is both simple and rapid, requiring only a brief handling period. This biosensor-based technology has been approved by the regulatory agency in Japan for in vitro diagnosis of Graves’ disease and is already routinely used in clinical laboratories^10^. Considering that V2R elevates intracellular cAMP concentrations via the Gs protein, this biosensor-based approach can be adapted for our purposes.

Using a biosensor-based approach for AVP measurement introduces two pivotal technical challenges. First, blood AVP levels are low in healthy individuals (in the picomolar range) and are even lower in patients with CDI^6^. This necessitates a highly sensitive assay to obtain precise measurements. Second, the synthetic peptide agonist desmopressin (DDAVP) is used to replace AVP to treat CDI^11^. Therefore, it is crucial to prevent V2R activation by AVP-mimetic peptides. In essence, a biosensor-based cAMP accumulation assay must possess sufficient sensitivity to assess minute AVP concentrations in the blood as well as selectivity to avoid activation by other therapeutic agonists.

In this study, to quantify plasma AVP concentration, we aimed to develop a simple and homogeneous assay employing a cAMP biosensor and modified receptor with improved sensitivity and selectivity.

## Results

### Screening of mammalian V2Rs for highly sensitive AVP measurements

**Figure 1:**
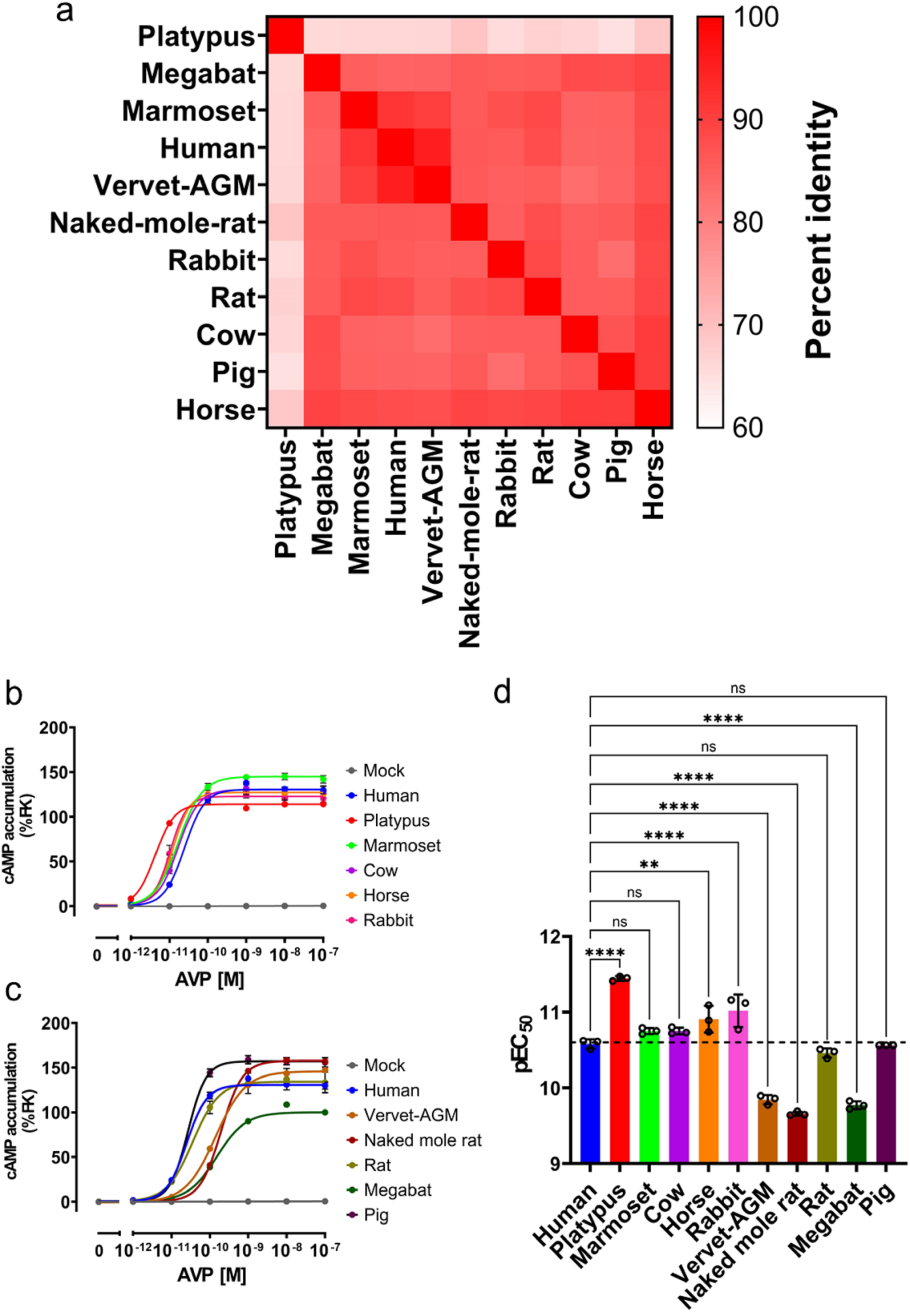
Screening of mammalian V2Rs for highly sensitive AVP assessment. (**a**) Percent identity matrix was constructed based on the amino acid sequences of the tested mammals. (**b**, **c**) Concentration–response curves of cyclic adenosine monophosphate accumulation assay using GloSensor-22F. (**b**) High pEC_50_ group results. (**c**) Low pEC_50_ group results. The data for mock cells and hV2R-expressing cells are shown in the two figures. Symbols and error bars represent the mean and standard error of the mean (SEM), respectively, of three independent experiments with each performed in duplicate. (**d**) The pEC_50_ values were calculated for each mammalian V2R based on the data from (**b**) and (**c**). Symbols and error bars represent the mean and SEM, respectively, of three independent experiments with each performed in duplicate. ****p < 0.001; **p < 0.005; *ns* not significant (A multiple comparison test was conducted using one-way analysis of variance with Dunnett’s post-hoc test).

To obtain a highly sensitive V2R-based sensor, we screened different mammalian V2Rs because non-mammals contain vasotocin instead of vasopressin^12^. We selected 10 mammalian V2Rs that are genetically distant from the human V2R. A phylogenetic tree developed based on amino acid sequences and represented the percent identity matrix of the amino acid sequences as heat map shows divergence of these V2R orthologs (Fig. 1a)^13^. Most mammals, including nine of the ten species selected, have the same AVP amino acid sequence as humans, while pigs have lysine–vasopressin, with one amino acid substitution at the eighth arginine of AVP. To begin functional studies, we transiently expressed each of the these mammalian V2Rs together with the luciferase-based GloSensor-22F cAMP reporter. The expression of all mammalian V2Rs was confirmed using the HiBiT-based cell-surface expression assay, with human V2R showing the highest expression among the receptors (Supplementary Fig. S1). We measured the increase in cAMP levels upon stimulation with titrated concentrations of AVP. The resulting bioluminescent signals were normalized to those of forskolin, an adenylyl cyclase activator, and fitted to a sigmoidal curve from which the negative logarithm of the half-maximal effective concentration (pEC_50_) was obtained (Fig. 1b,c, Supplementary Fig. S2a,b and Supplementary Table S1). Among the tested mammalian V2Rs, five V2Rs (platypus, marmoset, horse, cow, and rabbit) showed higher pEC_50_ values than the human V2R (hV2R) (Fig. 1d). Notably, the platypus V2R (pV2R) demonstrated a pEC_50_ value that was higher by approximately 0.8 (six-fold higher sensitivity on a linear scale) than that of hV2R (Supplementary Table S1). Thus, we chose pV2R as the potential receptor for measuring blood AVP concentrations.

### Modification of pV2R to reduce cross-reactivity with DDAVP

**Figure 2:**
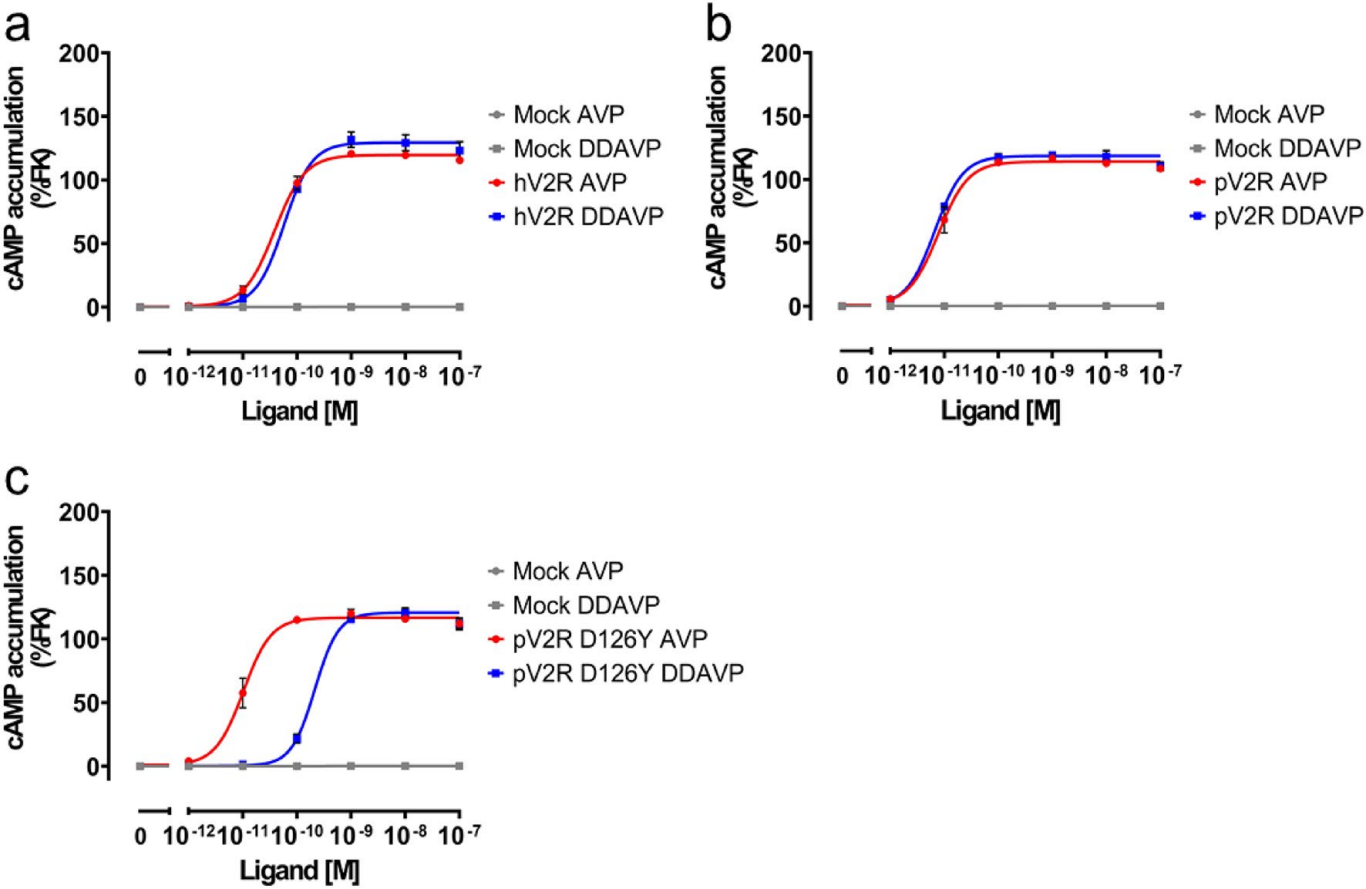
Modification of platypus V2R (pV2R) to reduce cross-reactivity with DDAVP. (**a**–**c**) Concentration–response curves of human V2R (**a**), wild-type pV2R (**b**), and pV2R D126Y (**c**) in response to AVP or DDAVP stimulation. In the wild-type pV2R, the curves for AVP and DDAVP stimulation overlap. However, in the pV2R D126Y, the curve for DDAVP stimulation shifted towards higher concentrations. Symbols and error bars represent the mean and standard error of the mean, respectively, of three independent experiments with each performed in duplicate.

To accurately measure AVP levels in patient-derived blood, it is imperative to eliminate cross-reactivity with DDAVP. Accordingly, we evaluated the cAMP response of pV2R to AVP and DDAVP stimulation. Notably, the cAMP response of pV2R to DDAVP was comparable to that of AVP, as observed for hV2R (Fig. 2a,b). This indicated the need to modify pV2R to prevent activation by DDAVP. To reduce cross-reactivity, we introduced a mutation in the ligand pocket of pV2R. A previous study found that the D103^2.68^Y (superscript numbers represent Ballesteros–Weinstein numbering) mutant of bovine V2R displayed a reduced affinity for DDAVP^14^. Leveraging this observation and the conservation of the 2.68 residue as aspartic acid in pV2R, we expressed the mutant pV2R (pV2R D126^2.68^Y) in HEK293A cells, with no significant difference in cell-surface expression levels between pV2R wild-type and D126Y (Supplementary Fig. S3). The mutant receptor exhibited a significantly reduced pEC50 value for DDAVP compared to that by the wild-type pV2R, while the response to AVP was equivalent to that of the wild-type pV2R, thereby improving the cross-reactivity by a difference of 1.3 between the pEC50 values for AVP and DDAVP (nearly 20-fold difference on a linear scale) (Fig. 2c and Table 1).

**Table 1:** The negative logarithms of half-maximal effective concentration (pEC_50_) values for AVP and DDAVP.

	*n* =	pEC_50_ (AVP)	pEC_50_ (DDAVP)
		Mean ± SEM	Mean ± SEM
hV2R	3	10.43 ± 0.09	10.24 ± 0.03
pV2R WT	3	11.12 ± 0.11	11.18 ± 0.04
pV2R D126Y	3	11.01 ± 0.11	9.66 ± 0.05

The pEC_50_ values were calculated based on the data shown in Fig. 2a–c.

### Measurement of AVP concentration in healthy human plasma

**Figure 3:**
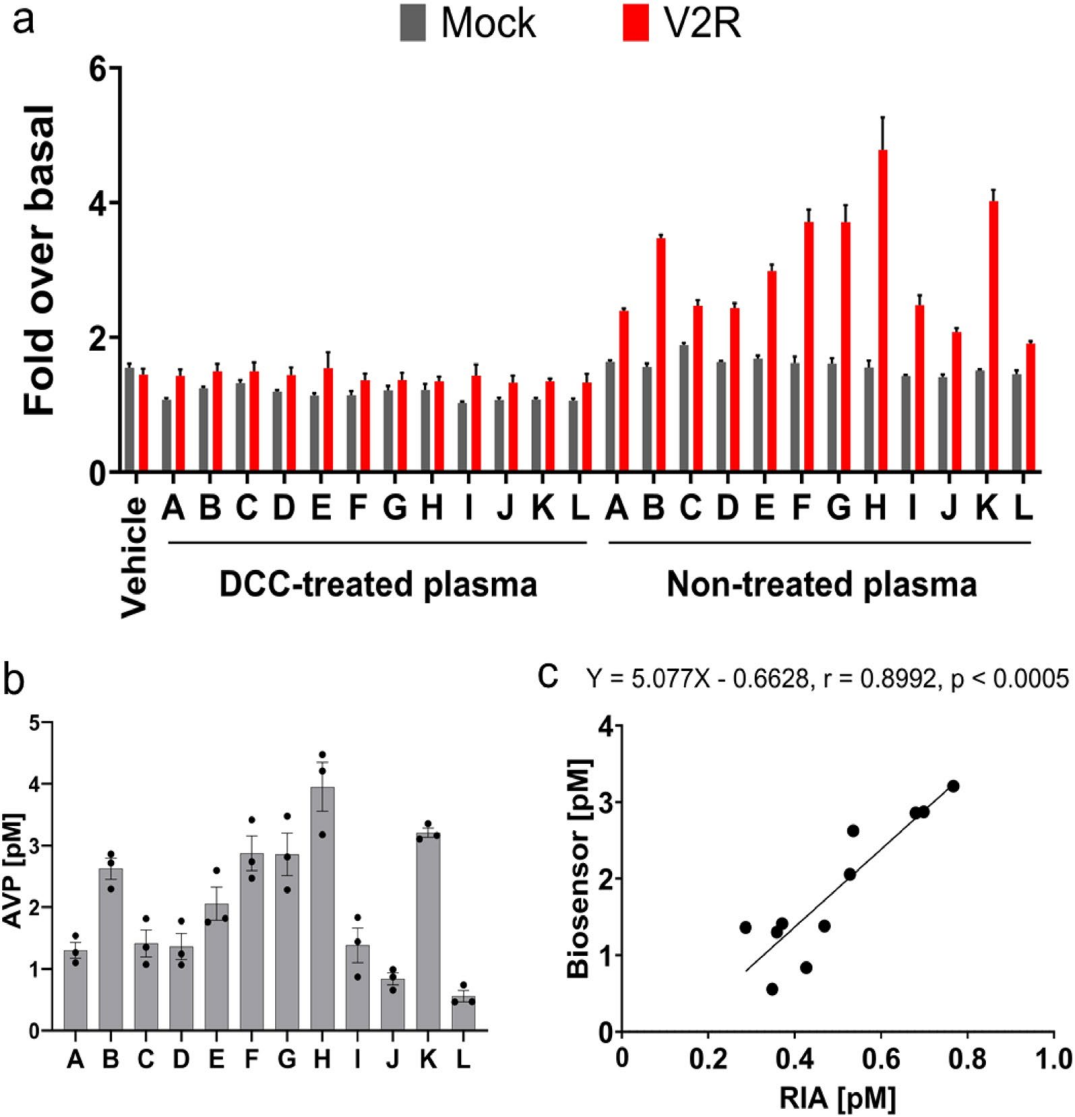
Measurement of AVP concentration in healthy human plasma. (**a**) Concentration of AVP in healthy human plasma was determined using the biosensor assay using pV2R D126Y and GloSensor-22F. The signal was obtained only under the conditions expressing the receptor, while the signal obtained under mock condition was comparable to that of vehicle. Symbols and error bars represent the mean and SEM, respectively, of three independent experiments with each performed in duplicate. (**b**) The plasma AVP concentration was quantified using serially diluted AVP as a standard solution. Symbols represent individual values, and error bars represent SEM based on three independent experiments with each performed in duplicate. (**c**) The plasma AVP concentration results obtained from the RIA and the biosensor-based assay were correlated. The human plasma was identical to that used in (**a**, **b**). The values obtained from the biosensor-based assay correspond to the results in (**b**).

We investigated the feasibility of using the pV2R D126Y mutant to measure plasma AVP concentration. Healthy human plasma samples (12; designated A–L) were subjected to a V2R–cAMP biosensor assay. Receptor-expressing cells exposed to the plasma samples showed higher cAMP accumulation than mock cells (no receptor control) or vehicle-treated cells (no plasma control) (Fig. 3a, Supplementary Tables S2 and S3). To prepare AVP-free plasma, the plasma samples were treated with dextran-coated charcoal (DCC). The DCC-treated plasma samples yielded signals comparable to those by the vehicle-treated cells (Fig. 3a and Supplementary Table S2), thus confirming that the luminescent signal was primarily attributable to small-molecule metabolites, including AVP, and not to larger molecules, such as the V2R-activating autoantibodies. These results strongly suggest that the luminescence signals mainly reflect an increase in intracellular cAMP concentration via the activation of V2R by AVP.

To quantify plasma AVP concentrations using the cAMP biosensor method, we measured cAMP responses using serially diluted AVP standards and calculated AVP concentrations in 12 healthy human plasma samples (Fig. 3b, Supplementary Fig. S4 and Supplementary Table S4). We found that AVP concentrations in human plasma samples ranged from 0.56 to 3.95 pM (mean ± standard deviation (SD): 2.0 ± 1.1 pM), which were in agreement with those reported previously (2.7 ± 1.4 pg/mL mean ± SD: 2.5 ± 1.3 pM)^7^. To determine the influence of off-target effects on the cAMP response, human plasma-induced cAMP responses were measured in mock-transfected cells. The signals exhibited by the mock cells were measured using the same AVP standards, resulting in AVP concentrations below 0.50 pM (Supplementary Fig. S5 and Supplementary Table S5). Subsequently, to confirm the influence of interfering substances in the plasma, sample-dilution tests were performed by diluting the plasma 2-, 4-, or 8-fold with the assay buffer and subjected to the biosensor-based assay. We found that, for about half of the samples (e.g., Plasma B, D, E, F, G, K), plasma concentration and luminescent signals were in a linear relationship, while in some samples (e.g., Plasma A, I, J, L), there was a tendency of saturating luminescent signals in higher plasma concentrations (Supplementary Fig. S4). The occurrence of the relatively poor linearity in some samples suggests potential disturbances caused by assay-interfering substances in the plasma samples. Additionally, for the same plasma samples, we assessed the plasma AVP concentrations using the RIA-based method, which is a standard in vitro diagnostic technique (Supplementary Table S6). We observed a strong correlation (Pearson's correlation value: 0.90) between the V2R-cAMP biosensor and RIA assay results, although the estimated AVP concentrations were higher for the cAMP biosensor assay, by approximately five-fold, than for the RIA-based method (Fig. 3c). Despite occurrences of the dilution-linearity issue in the biosensor assay (Supplementary Fig. S4), these benchmarking experiments show that the performance of this assay is comparable to that of RIA, a clinically used assay. Our findings suggest that the biosensor-based assay developed in this study is not only simple but also useful for measuring plasma AVP concentrations.

## Discussion

The biosensor-based assay simplifies the assay procedure and shortens the assay time. Notably, the V2R-cAMP biosensor assay can measure plasma AVP concentrations in approximately 2 h, as opposed to the 3-day duration required for the RIA method^15^. While RIA requires the extraction of AVP from the plasma, our assay is homogeneous and does not require time-consuming procedures^7,8^. The assay developed in this study requires time for cell seeding and transfection, but we believe that this problem can be solved using cryopreserved cells, as reported previously^9,10^. These findings demonstrate the potential of this simplified assay for the quantification of plasma AVP. Clinically used DDAVP concentrations are tolerable for the biosensor-based assay with the modified receptor established in this study. The maximum concentration of DDAVP in the blood of patients with CDI treated with this drug was approximately 30 pM^16^. Our analysis indicates that 30 pM of DDAVP generates a cAMP signal equivalent to 0.4 pM of AVP (Fig. 2c). In healthy individuals, 0.4 pM is within the lower range of plasma AVP concentrations. Furthermore, the concentration of DDAVP in blood was estimated to be less than 30 pM^17^. Based on these observations, we concluded that the cross-reactivity with DDAVP was minimal. Thus, a biosensor-based assay using pV2R D126Y is suitable for measuring plasma AVP concentrations and offers sufficient sensitivity and specificity. We will generate additional mutants that may enable us to generate a receptor with high sensitivity to AVP and upgrade our assay in the future.

Nonspecific reactions are often a problem in assays that use biological samples, including plasma. Human plasma contains myriad GPCR ligands that may activate endogenous GPCRs, thereby elevating intracellular cAMP levels. HEK293A cells used in this study express numerous GPCRs^18^. In the control experiments using the mock cells (untransfected with the cAMP GloSensor plasmid alone), the signal induced by the plasma sample C was significantly higher than that induced by vehicle treatment alone (Fig. 3a and Supplementary Table S3). The GloSensor response in receptor-expressing cells was higher than that in mock cells, and the plasma AVP concentration in mock cells was near the lower limit in healthy individuals (Supplementary Fig. S5 and Table S5). These results indicate that an increase in intracellular cAMP concentrations, potentially attributable to the activation of other endogenous GPCRs by ligands in the plasma, had a minimal effect on the accuracy of AVP measurement by our assay. We note, however, that poor dilution linearity was observed in certain plasma samples (Supplementary Fig. S4), which may indicate the presence of in the plasma that interfere with the assay. The plasma for this study was obtained commercially, and consequently, details about its origin and any prior drug treatments are unclear. Employing plasma with well-documented background information, ideally collected in a controlled hospital setting, would offer valuable insights into potential interference factors. Resolving this issue is crucial for the clinical implementation of the biosensor-based assay.

The biosensor-based assay developed in this study showed a strong correlation with RIA. However, the estimations by the biosensor-based assay did not align perfectly with those by RIA, with the slope of the regression line being 5.077, thus indicating that the estimation by the biosensor-based assay tended to be approximately five times higher than that by RIA (Fig. 3c). These discrepancies could be attributed to the AVP conditions during the assays. In RIA, AVP is extracted from plasma samples using organic solvents, whereby substances hindering antigen–antibody reactions are eliminated^15^. In blood, AVP binds to the carrier protein NP2; however, in RIA, the carrier protein undergoes denaturation, thus resulting in carrier-protein-free AVP^19^. In the biosensor-based assay, plasma samples were directly applied to V2R-expressing cells, providing AVP bound to the carrier protein during measurement. In this study, we used synthetic AVP as the standard for biosensor-based assay, and the diluted standard solution prepared using synthetic AVP may differ from physiological conditions. Given the possibility that the biosensor-based assay and RIA may evaluate distinct states of AVP, we speculated that the variation in the condition of the AVP measured contributed to the observed discrepancies in the plasma AVP concentrations calculated by the two methods. Although the calculated values may differ between RIA and the biosensor-based assay owing to variations in the AVP status, there is a strong correlation between the two methods. This suggests that the biosensor-based assay is a promising alternative to RIA. Recently, the LC–MS/MS method has been developed to measure AVP levels, which has excellent sensitivity and selectivity. LC–MS/MS also shows a strong correlation with RIA; however, there is a discrepancy between the values obtained from the two methods^20,21^. Specifically, LC–MS/MS tends to yield lower values compared to RIA. Despite high correlations among RIA, the biosensor-based assay, and LC–MS/MS, their values do not perfectly align^21,22^. The discrepancies in values among these three methods may warrant examination in the future. Due to the unavailability of CDI patient specimens in this study, our investigation was confined to specimens obtained from healthy individuals. To assess the clinical performance of the biosensor-based assay, future studies are warranted to examine CDI patient specimens.

A cell-free assay offers an alternative approach to the biosensor-based assay. Recently, a technique for measuring GPCR activity using conformation-specific binders and lysates from GPCR-expressing cells has been reported^23^. Although challenges such as lower sensitivity compared to cell-based assays have been noted, potential improvements in the future may enable the construction of a cell-free assay using the receptors developed in this study. If these challenges are overcome, a cell-free assay would offer simpler and more reproducible measurements than a live cell-based assay.

In conclusion, this study has established a method for measuring plasma AVP concentrations using a cAMP biosensor. In addition to simple and quick measurements, the elimination of radioisotopes reduces the burden on workers and minimizes environmental impact. In the future, we intend to conduct clinical trials to apply this assay in diagnosing CDI and expect it to contribute to the improvement of CDI diagnosis.

## Methods

### Plasmid

All V2R genes were human codon-optimized and synthesized by GenScript. The V2R constructs were fused to the HA signal sequence, followed by a FLAG-linker-HiBiT-linker sequence (MKTIIALSYIFCLVFADYKDDDDKGGSGGGGSGGSSSGGGVSGWRLFKKISGGSGGGGSG), and cloned into the pCAGGS vector (a kind gift from Dr. Jun-ichi Miyazaki at Osaka University, Japan). The GloSensor-22F cAMP biosensor gene was human codon-optimized and synthesized by GenScript, and encoded into the pCAGGS plasmid.

### Cell culture

HEK293A cells (Thermo Fisher Scientific, USA) were maintained in Dulbecco’s Modified Eagle’s Medium (DMEM, Nissui Pharmaceutical, Japan) supplemented with 10% fetal calf serum (GIBCO, USA), 100 U/mL penicillin, 100 μg/mL streptomycin, and 200 mM l-glutamine (FUJIFILM Wako Pure Chemical Corporation, Japan) (complete DMEM). The cells were maintained in a humidified incubator with 5% CO_2_ at 37 °C.

### Transfection

Plasmid transfection was performed using polyethyleneimine (PEI MAX; Polyscience, USA) as the transfection reagent. The cells were seeded in a 6-well plate at a density of 4.0 × 10^5^ cells per well with 2 mL of the complete DMEM and cultured for 1 day. For transfection, a polyethyleneimine solution was prepared by mixing 5 μL of 1 mg/mL polyethyleneimine with 95 μL of Opti-MEM (Thermo Fisher Scientific). The V2R-encoded plasmid (200 ng) and the GloSensor-22F-encoded plasmid (1 μg) were diluted in 100 μL of Opti-MEM. A negative control in which V2R plasmid was replaced with an empty pCAGGS plasmid was referred to as mock. The polyethyleneimine solution and the plasmids were combined and incubated for 20 min at room temperature. The resultant polyethyleneimine–plasmid DNA complex was added to the cells, and the cells were cultured for 1 day before performing the assay.

### GloSensor-based cAMP accumulation assay

GloSensor-based cAMP accumulation assay was performed as previously described, with minor modifications^23^. Transfected cells were harvested in phosphate-buffered saline containing 1 mM EDTA, followed by centrifugation at 190×*g* for 5 min. The cell pellet was suspended in the assay buffer consisting of Hanks' balanced salt solution containing 0.01% bovine serum albumin (BSA) and 5 mM HEPES at pH 7.4 (assay buffer). Next, the cells were seeded in white 96-well plates at a volume of 40 μL per well, and 10 μL of d-Luciferin solution (10 mM d-Luciferin potassium salt diluted in the assay buffer) was added to each well. After 2 h of incubation in the dark at room temperature, the initial luminescent counts were measured using the SpectraMax L plate reader (Molecular Devices, USA) or GloMax Explorer (Promega Corporation, USA). Subsequently, the cells were treated with either 10 µL of the vehicle (assay buffer), AVP (Peptide Institute, Japan), or DDAVP (Peptide Institute), at concentrations ranging from 1 pM to 100 nM, as well as 10 μM forskolin (FUJIFILM Wako Pure Chemical). Note that, in the case of plasma AVP concentration measurement, human plasma were added to 10 µL each (total assay volume of 60 µL). Thereafter, the kinetics of the assay were measured for 20 min, and the fold-over basal value was calculated from the mean of 18–20 min after compound addition. The fold-over basal values were normalized to those obtained for the forskolin-treated group. Representative luminescence kinetics are shown in Supplementary Fig. S7.

### HiBiT-based cell-surface expression assay

The transfected cells used for the GloSensor-based cAMP assay were also used for the HiBiT-based cell-surface expression assay. The cells were seeded in white 96-well plates at 5 μL per well, and 45 μL of the assay buffer was added to each well. Next, 50 μL of substrate buffer (1:200 LgBiT stock solution and 20 μM furimazine in the assay buffer) was added to each well and gently mixed by tapping. After 40 min of incubation at room temperature, the luminescence signal was measured using the luminescent plate reader for 5 min. The means of the luminescent signals over 5 min were calculated and normalized to those of hV2R-expressing cells.

### Multiple sequence alignment and percent identity matrix

Multiple sequence alignment and percent identity matrices based on the amino acid sequences of the tested mammalian V2Rs were ascertained using Clustal Omega (https://www.ebi.ac.uk/Tools/msa/clustalo/)^24,25^. The amino acid sequences were obtained from a previous study^13^. The amino acid sequence alignment between platypus pV2R and hV2R are shown in Supplementary Fig. S8 and a number of key residues in this study are marked. The percentage identity matrix data obtained were visualized as a heat map using GraphPad Prism 9 (GraphPad).

### Removal of AVP from human plasma using DCC treatment

DCC treatment was performed using a previously reported method with modifications^26^. A DCC solution was prepared by combining 5% activated charcoal (Fujifilm Wako Pure Chemical), 0.3% dextran (Sigma-Aldrich), and 2% BSA (Seracare) in distilled water with gentle stirring. The resulting DCC solution was further aliquoted into 1.5 mL tubes, with each tube containing 500 μL of the solution. The tubes were centrifuged at 4000×*g* for 5 min to remove the supernatant. Subsequently, 500 μL of human plasma was added to the DCC precipitate and gently stirred at room temperature for 1 h. After incubation, the tubes were further centrifuged at 4000×*g* for 5 min. The resulting supernatant was filtered using a 0.22 µm syringe filter to obtain the AVP-free plasma sample.

### Measurement of plasma AVP concentrations using RIA

All human plasma samples were procured from Access Biologicals Inc. Plasma AVP concentrations were measured using a commercial RIA kit (AVP kit YAMASA, YAMASA Corporation), according to the manufacturer’s instructions. In brief, the standard solutions or ethanol-extracted human plasma were initially treated with primary antibodies at 4 °C for 1 day. Next, reactions with 125I-labeled AVP were performed for 1 day, following which secondary antibodies and PEG solution were added and incubated at 4 °C for 4 h. Finally, the supernatant was removed by centrifugation, and radioactivity was measured using a gamma counter (PerkinElmer, USA).

### Data analysis

Concentration–response curves for all data were fitted to a nonlinear regression: variable slope (four-parameter) function and absolute Hill slope values less than 1.5 were set in GraphPad Prism 9 (GraphPad, UK), and negative logarithms of the half-maximal effective concentration (pEC_50_) values were obtained. Replicates of each experiment are shown in the figure legends. A multiple comparison test was conducted using one-way analysis of variance with Dunnett’s post-hoc test, in which *P-*value ≤ 0.05 indicates a statistically significant difference.

### Supplementary Information

Supplementary Information 1.

Supplementary Information 2.

## Data Availability

The data used in this study are available from the corresponding author upon reasonable request.
