# Supplementary material for: A cAMP-biosensor-based assay for measuring plasma arginine–vasopressin levels
Source: Sci Rep. 2024 Apr 24;14:9453. doi: 10.1038/s41598-024-60035-4 (PMC11043374; doi:10.1038/s41598-024-60035-4)
Supplement: Supplementary file 1 — Supplementary Information 1. [file 41598_2024_60035_MOESM1_ESM.pdf]

Supplementary Table S1. pEC_{50} values of the mammalian V2Rs shown in Figure 1d.

Origin of V2R species	*n* =	pEC_{50}	*P* value vs human	
Human	3	10.58 ± 0.04		
Platypus	3	11.44 ± 0.02	<0.001	****
Marmoset	3	10.75 ± 0.03	0.209	ns
Vervet-AGM	3	9.84 ± 0.04	<0.001	****
Naked mole rat	3	9.66 ± 0.02	<0.001	****
Rat	3	10.46 ± 0.04	0.562	ns
Megabat	3	9.77 ± 0.04	<0.001	****
Pig	3	10.56 ± 0.00	>0.999	ns
Cow	3	10.75 ± 0.03	0.209	ns
Horse	3	10.91 ± 0.12	0.002	**
Rabbit	3	11.02 ± 0.15	<0.001	****

The pEC_{50} values were obtained from three independent experiments, each performed in duplicate. *P* values were calculated using a one-way analysis of variance. *P* values were calculated using Dunnett's multiple comparison test. **, $P < 0.005$; ****, $P < 0.001$; ns, not significant.

Supplementary Table S2. Fold-over basal values for the V2R condition in Figure 3a.

		Fold over basal Mean ± SEM	*P* value vs vehicle	
Vehicle		1.45 ± 0.07		
DCC-treated plasma	A	1.43 ± 0.06	>0.9999	ns
	B	1.50 ± 0.07	>0.9999	ns
	C	1.50 ± 0.08	>0.9999	ns
	D	1.44 ± 0.07	>0.9999	ns
	E	1.54 ± 0.14	>0.9999	ns
	F	1.36 ± 0.07	>0.9999	ns
	G	1.37 ± 0.07	>0.9999	ns
	H	1.35 ± 0.05	>0.9999	ns
	I	1.43 ± 0.11	>0.9999	ns
	J	1.33 ± 0.07	>0.9999	ns
	K	1.35 ± 0.03	>0.9999	ns
	L	1.33 ± 0.08	>0.9999	ns
Non-treated plasma	A	2.39 ± 0.02	0.0015	**
	B	3.47 ± 0.07	<0.0001	****
	C	2.47 ± 0.06	0.0005	***
	D	2.43 ± 0.05	0.0009	***
	E	2.98 ± 0.07	<0.0001	****
	F	3.71 ± 0.13	<0.0001	****
	G	3.71 ± 0.17	<0.0001	****
	H	4.78 ± 0.31	<0.0001	****
	I	2.48 ± 0.12	0.0005	***
	J	2.08 ± 0.05	0.0838	ns
	K	4.02 ± 0.12	<0.0001	****
	L	1.91 ± 0.05	0.3877	ns

Mean ± standard error of the mean of fold-over basal values were obtained from three independent experiments with each preformed in duplicate. *P* values were calculated using Dunnett's multiple comparison test. *, $P < 0.05$; **, $P < 0.005$; ***, $P < 0.001$; ****, $P < 0.0001$; ns, not significant.

Supplementary Table S3. Fold-over basal values for the mock condition in Figure 3a.

		Fold over basal Mean ± SEM	*P* value vs vehicle	
Vehicle		1.55 ± 0.04		
DCC-treated plasma	A	1.08 ± 0.02	<0.0001	****
	B	1.24 ± 0.03	0.002	**
	C	1.32 ± 0.04	0.0407	*
	D	1.20 ± 0.03	0.0002	***
	E	1.14 ± 0.21	<0.0001	****
	F	1.15 ± 0.39	<0.0001	****
	G	1.21 ± 0.04	0.0005	***
	H	1.22 ± 0.06	0.0007	***
	I	1.03 ± 0.02	<0.0001	****
	J	1.07 ± 0.03	<0.0001	****
	K	1.08 ± 0.03	<0.0001	****
	L	1.06 ± 0.04	<0.0001	****
Non-treated plasma	A	1.64 ± 0.02	0.9556	ns
	B	1.57 ± 0.04	>0.9999	ns
	C	1.89 ± 0.04	0.0005	***
	D	1.63 ± 0.01	0.9694	ns
	E	1.68 ± 0.03	0.5361	ns
	F	1.62 ± 0.06	0.9932	ns
	G	1.62 ± 0.06	0.9982	ns
	H	1.55 ± 0.07	>0.9999	ns
	I	1.43 ± 0.02	0.6727	ns
	J	1.41 ± 0.03	0.4987	ns
	K	1.51 ± 0.10	>0.9999	ns
	L	1.46 ± 0.04	0.922	ns

Mean ± standard error of the mean of fold-over basal values were obtained from three independent experiments with each preformed in duplicate. *P* values were calculated using Dunnett's multiple comparison test. *, $P < 0.05$; **, $P < 0.005$; ***, $P < 0.001$; ****, $P < 0.0001$; ns, not significant.

Supplementary Table S4. Plasma AVP concentrations measured using the biosensor-based assay shown in Figure 3b and 3c.

	AVP [pM] Mean ± SEM
A	1.30 ± 0.13
B	2.62 ± 0.17
C	1.41 ± 0.22
D	1.36 ± 0.21
E	2.06 ± 0.27
F	2.87 ± 0.28
G	2.86 ± 0.35
H	3.95 ± 0.40
I	1.38 ± 0.28
J	0.84 ± 0.10
K	3.21 ± 0.08
L	0.56 ± 0.09

Mean and standard error of the mean of plasma AVP concentrations were obtained from three independent experiments each performed in duplicate.

Supplementary Table S5. Plasma AVP concentrations from mock cells measured by the biosensor-based assay shown in Supplementary Figure S5.

	AVP [pM]		
	Experiment		
Sample ID	#1	#2	#3
A	NA	0.11	0.09
B	NA	0.08	NA
C	0.46	0.54	0.50
D	NA	0.09	NA
E	NA	0.11	0.35
F	NA	NA	0.45
G	NA	0.04	0.34
H	NA	NA	0.31
I	NA	NA	NA
J	NA	NA	NA
K	NA	NA	NA
L	NA	NA	NA

Plasma AVP concentrations were obtained from three independent experiments, each performed in duplicate. NA, not available due to values below the limit of quantification.

Supplementary Table S6. Plasma AVP concentrations measured using RIA shown in Figure 3c.

	AVP [pM] Mean ± SEM
A	0.36 ± 0.01
B	0.54 ± 0.04
C	0.37 ± 0.06
D	0.29 ± 0.04
E	0.53 ± 0.03
F	0.70 ± 0.04
G	0.68 ± 0.05
H	ND
I	0.47 ± 0.07
J	0.43 ± 0.04
K	0.77 ± 0.07
L	0.35 ± 0.03

Three independent experiments were performed in duplicate. ND, no data due to insufficient sample volumes for measurement.

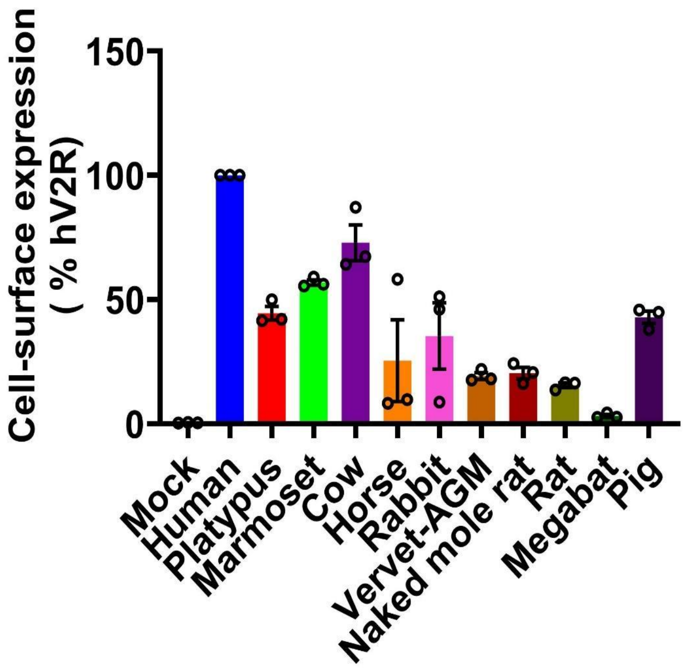

Supplementary Figure S1. Cell-surface expression levels of 11 mammalian V2Rs

Expression levels were determined using the HiBiT-based cell-surface expression assay. Symbols and error bars represent the mean and standard error of the mean, respectively, of three independent experiments with each performed in duplicate.

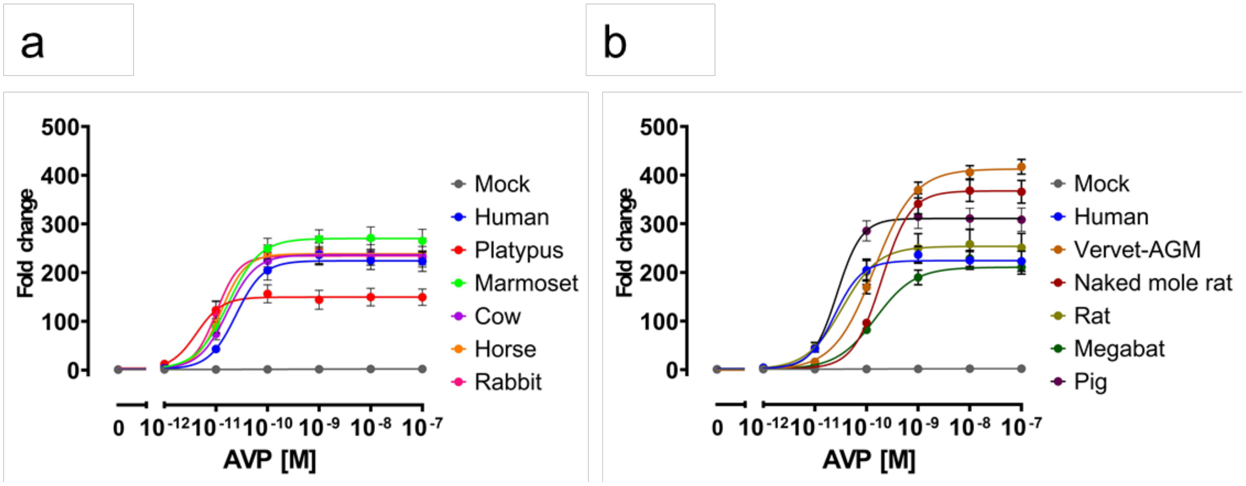

Supplementary Figure S2. Concentration-response curves of the biosensor assay.

Fold-change values of the same dataset in Fig. 1b and 1c, but without standardization to the forskolin-treated condition, are illustrated. (**a**) High pEC_{50} group results and (**b**) low pEC_{50} group results. For the mock and human data, the same dataset is shown in the two figure panels. Symbols and error bars represent the mean and the standard error of the mean, respectively, of three independent experiments with each performed in duplicate.

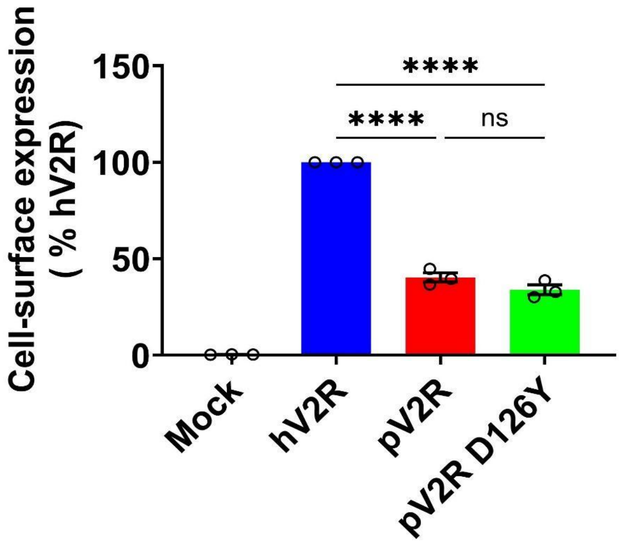

Supplementary Fig. S3. Cell-surface expression levels of selected V2R constructs.

Expression levels of human V2R, platypus V2R and platypus V2R with the D126Y mutation were measured using the HiBiT-based cell-surface expression assay. Symbols and error bars represent the mean and standard error of the mean, respectively, of three independent experiments with each performed in duplicate. *P* values were calculated using a one-way analysis of variance with Dunnett's post-hoc test. ****, $p < 0.0001$; ns, not significant.

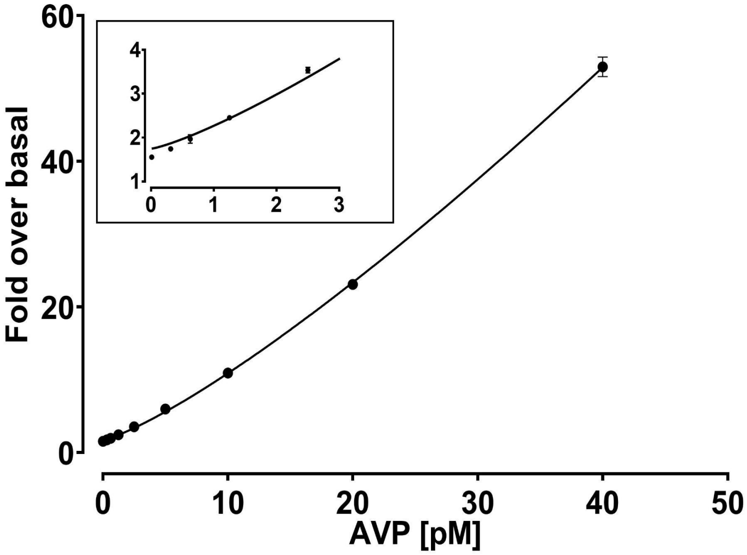

Supplementary Figure S4. Representative standard curve of the biosensor-based assay. Symbols and error bars represent the mean and standard deviation, respectively, with each performed in duplicate as biological replicates.

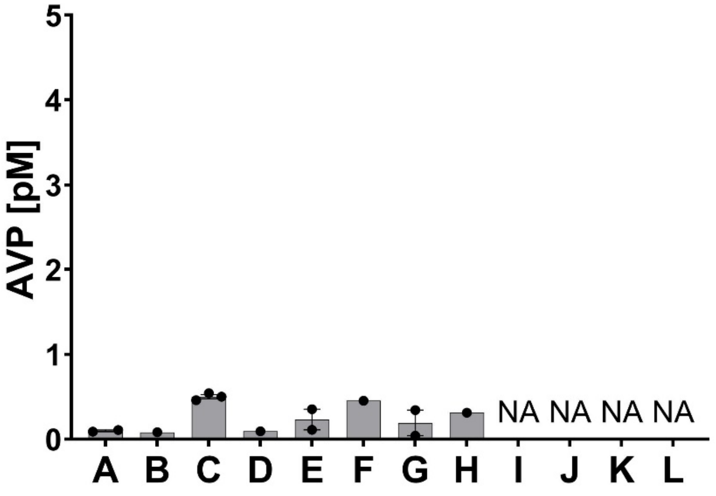

Supplementary Figure S5. Minimal effects of human plasma samples in the mock condition. The luminescent signals detected in the mock-transfected (V2R-minus, but cAMP GloSensor-22F-expressing) cells were putatively calculated into AVP concentrations using pV2R D126Y-expressing HEK293 cells and their responses to serially diluted AVP as a standard solution. Symbols represent individual values, and error bars represent the standard error of the mean based on three independent experiments with each performed in duplicate. The calculated values are shown in Supplementary Table S5, and the values below the limit of lower quantifications are not presented in this figure. NA, not available due to values below the limit of quantification.

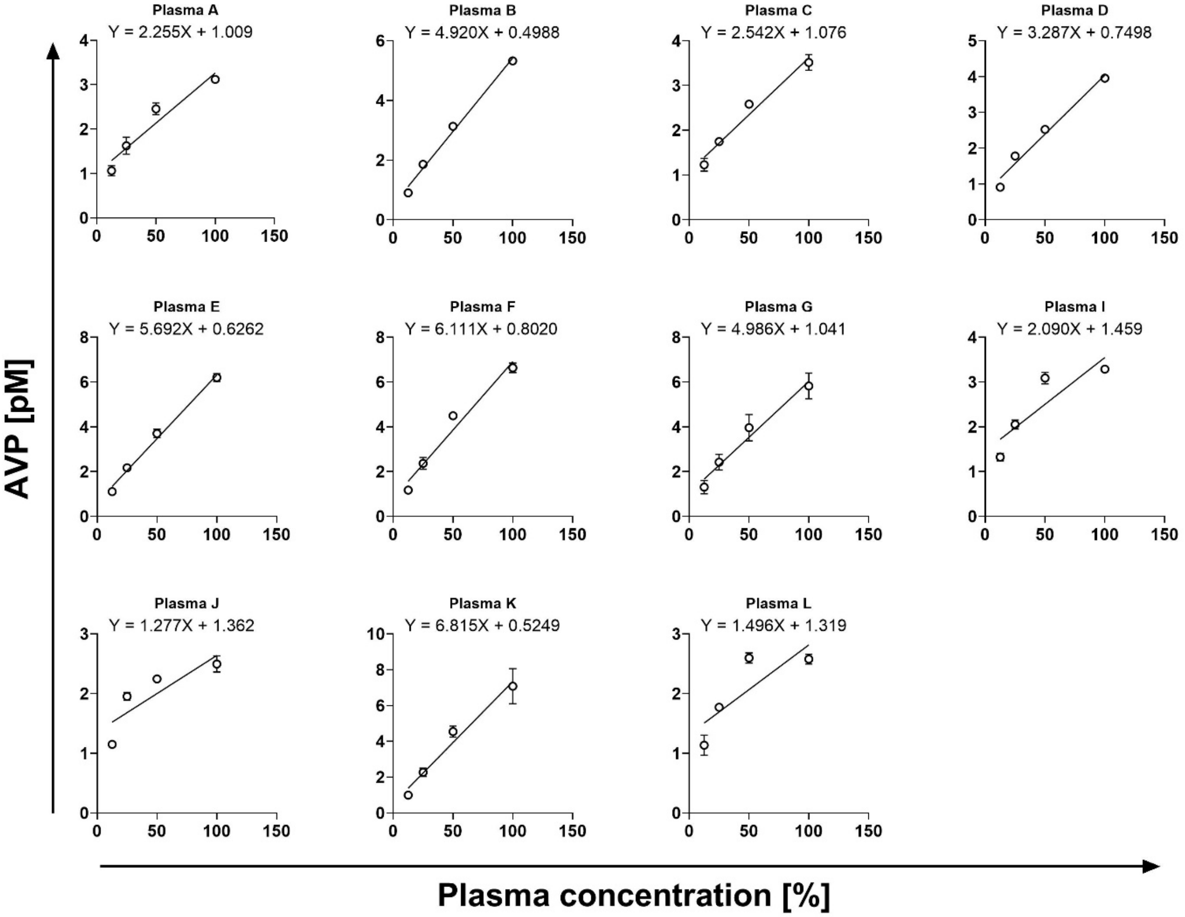

Supplementary Figure S6. Sample-dilution test of the biosensor-based assay.

The human plasma samples A-L were diluted by a factor of 2, 4, or 8 with the assay buffer and measured using the biosensor-based assay. Plasma concentration represents the dilution factor of plasma; 2-, 4-, and 8-fold dilutions correspond to 50, 25, and 12.5%, respectively; the plasma concentration of 100% indicates no dilution. Symbols and error bars represent the mean and the standard deviation, respectively, with each performed in duplicate as biological replicates. Note that, due to the insufficient volume of the sample H, its measurement had not been done.

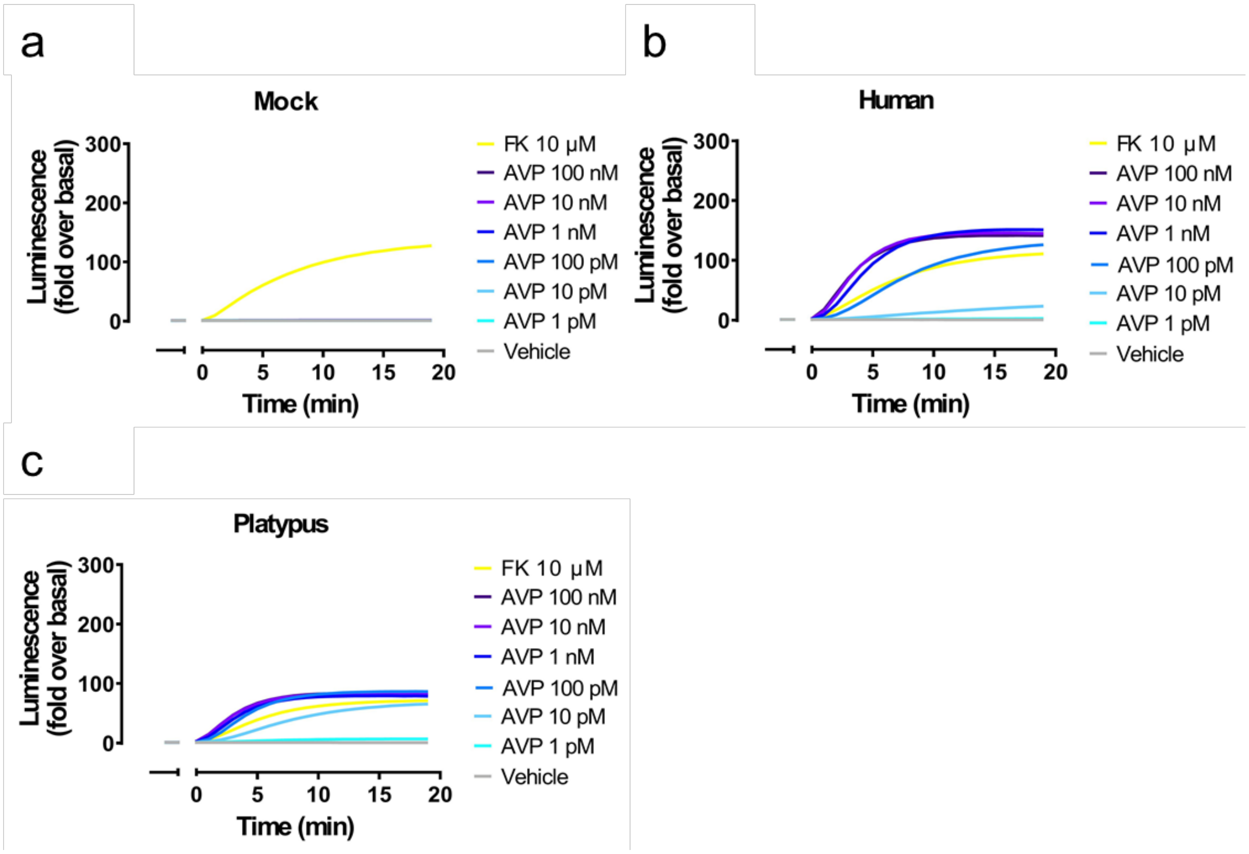

Supplementary Figure S7. Representative luminescence kinetics.

Representative luminescence kinetics of mock cells **(a)**, human V2R-expressing cells **(b)**, and platypus V2R-expressing cells **(c)** to serially diluted AVP concentrations in the biosensor-based assay. FK, forskolin. Luminescence kinetic curves represent the mean of duplicates as biological replicates for each ligand.

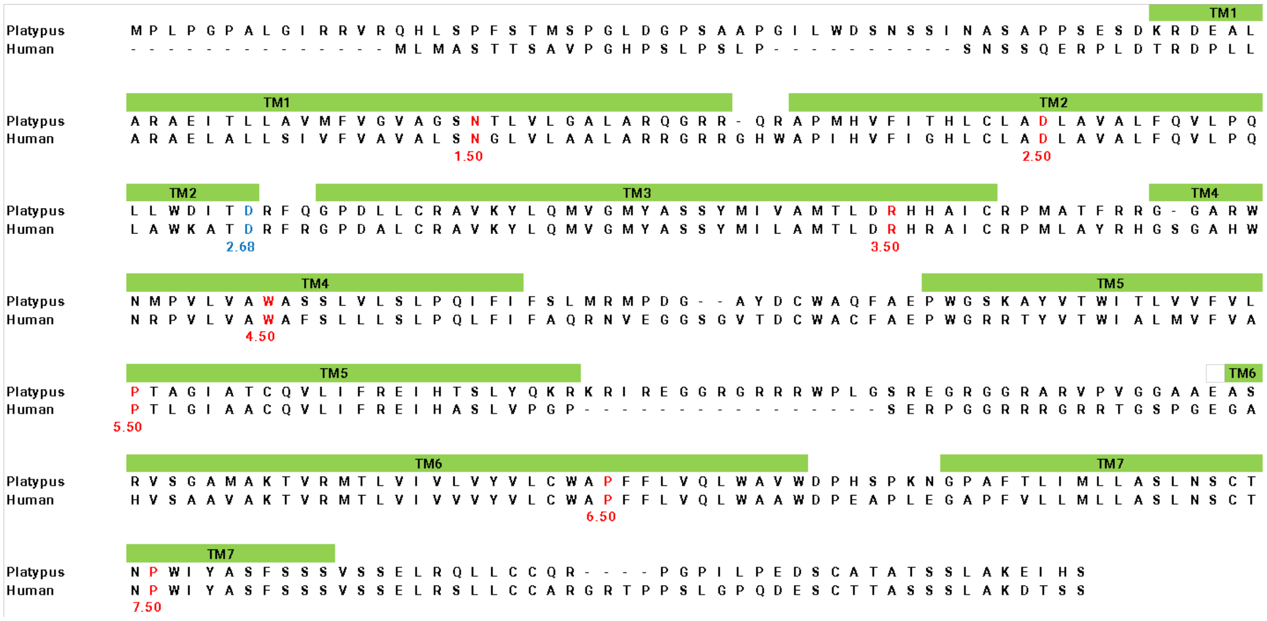

Supplementary Figure S8. Sequence alignment between platypus V2R and human V2R. The green ribbon represents the transmembrane domain. Blue letters represent the amino acid residue $D^{2.68}$, which corresponds to D126 in pV2R. Red letters represent the most conserved residues in each transmembrane domain and their Ballesteros–Weinstein numbers are shown at the bottom.
